# Walls of resistance: Underground memories and political violence in Colombia

**DOI:** 10.1080/02757206.2023.2248166

**Published:** 2023-08-25

**Authors:** Patrick Naef

**Affiliations:** Department of Geography and Environment, University of Geneva, Geneva, Switzerland

**Keywords:** Political violence, memory, resistance, murals and street art, Colombia

## Abstract

In this article, I examine ‘underground memories’ to demonstrate how they serve as resources for resistance in the margins of Colombia. I focus on their relations with the urban fabric, looking at the ways the walls of Bogota and Medellin are used as canvases for spreading images and narratives about the conflict. I suggest that murals representing the violence serve as a repository for memories; they challenge hegemonic narratives and contribute to the recovery of public space. This analysis draws on three case studies. In the first one, I examine the impact of a mural in Bogota that denounced extrajudicial killings involving the Colombian army. The second case focuses on a community initiative aimed at collecting testimonies from residents in a marginalized district of Medellin. Finally, the last case study analyses the touristification of some of the many murals depicting the violence in Medellin. I argue that, to different degrees, all the memorial projects presented in this study challenge state narratives. Through representations of murdered teenagers, suspect military officers and even drug cartel bosses, they raise questions of social justice, impunity, illegality and the dramatic banalization of violence in the country. They broaden the narrative on the recent past, through histories and images that the Colombian state is still reluctant to unearth.

## Introduction

Colombia saw an explosion of memorial projects in the last decade. Some, like public museums and historical reports, were initiated by the state, while others emerged within communities impacted by the violence. This surge of memory occurred in a disputed context, where memorial entrepreneurs often relayed conflicted representations of past violence. The Colombian state was itself an important victimizer. Thus, even if public authorities have started to acknowledge their part of responsibility for the conflict, many collectives of victims are still struggling to shed light on the political violence that affected, and continues to impact, their country. In this contested arena, shaped by impunity, domination and silence, memory work offers a means for individuals and groups in the margins of Colombia to resist some of the hegemonic discourses and representations of the armed conflict and the ensuing peace. It provides a way of making their voices heard and of becoming actors in the recollection of their past and the construction of their future.

In this article, I examine ‘underground memories’ originating in Colombia’s margins. They are generally contested, disregarded in mainstream political discourses, and overlooked in public institutions. Moreover, I focus on their relations with the urban fabric. I look at the ways the walls in Bogota, the capital, and Medellin, the second city of the country, are used as canvases for spreading images and narratives about the violence. I analyze the production and impact of murals in both cities, considering them in some ways as alternative history textbooks. I suggest that not only do they serve as a repository for memories in these marginal communities, but also that they facilitate the recovery of public space. They challenge a hegemonic set of narratives and representations on the conflict in Colombia, while also reconnecting residents with the street, contributing to community gatherings and political debates in the city. In sum, I argue that memories in the margins of cities like Bogota and Medellin, beyond the conservation of the past, strengthen residents’ rights to their city.

In keeping with the central topic of this special issue, I am interested in the ways some of these underground memories are (un)earthed. I address the legacy of political violence in Colombia in order to show how it reverberates in the present. Adopting a bottom-up perspective and considering mainly neighbourhood mobilizations, I look at the (un)earthings of violence through symbolic, aesthetic, material and discursive processes, to expose how they affect current political and social contexts. Three case studies are developed below. In the first one, I examine the impact of a mural in Bogota that denounced extrajudicial killings involving the Colombian army. I will demonstrate how its covering up and attempts from high-ranking officers to erase it from social media paradoxically contributed to widely diffusing its message. The second case focuses on a community initiative aimed at collecting testimonies from residents in a peripheral district of Medellin. These neighbourhood stories are represented in murals painted on the walls of a cemetery: they feature portrayals of murdered teenagers, critical representations of the state and pictures of human rights activists. Finally, the last case study analyzes the touristification of some of the many murals depicting the violence in Medellin. It shows how the success of tourism and street art in the second city of Colombia helped depoliticize the messaging that was initially spread by artists and tour guides.

As stated in the introduction to this special issue, memory is considered as travelling and moving across space and time. Scholars in memory studies are expressing a growing interest in the multiple scales in which memory operates. This article hopes to contribute to this debate, by offering an exploration of the ways underground memories travel among these different contexts, but also by looking at the consequences of their journeys. Henry Rousso (2007) has long stressed the importance of looking beyond the national level in order to understand memorial phenomena. States are increasingly confronted with competing visions of the past, which challenge the traditional domination of national memory. As Rousso commented, political systems in recent decades have led to the formation of a new public space, at national, regional and global levels. This space is characterized by a surge of groups proposing historical narratives that tend to reject national versions of history, suspected of blindness and censure. He adds that these discourses tend to abolish the traditional borders between the scientist, the politician, the actor, the activist, opening a plurality of interpretations of the past. Hence, by looking specifically at the walls and streets of the two main cities of Colombia, this article will explore the possibilities that ‘traveling memories’ open up, but also the limits imposed on their journeys. Indeed, I maintain that if collective (and underground) memory provides resources for resistance, it can also include dispossession processes. As the example of tourism will demonstrate, street art certainly gave memorial entrepreneurs (the artists producing the murals and the tour guides presenting them) a space for activism and resistance. However, some of my findings suggest that the commodification that accompanied tourism and the take-over of the sector by criminal structures led to a depoliticization of the narratives.

This contribution is part of a larger research project exploring urban violence, memory and resistance. Results were collected during several phases of fieldwork conducted between January 2019 and October 2022. During this period, I carried out approximately a hundred interviews with human rights defenders; tourism entrepreneurs; artists; government officials; former paramilitaries and gang members; residents and business owners in Colombia’s *barrios populares.* The first part of this article offers some theoretical insights into the ways memorial practices and discourses serve as resistance, especially in the form that Michael Pollak (2006) considers as ‘underground memory’. The second part presents the three case studies, with the objective of understanding better how underground memories travel in the disputed memorial arena of Colombia. A discussion finally examines the implications of (un)earthling political violence in the current and disputed context of Colombia.

## Memory as a resource for resistance in Latin America

Questions of domination and uneven access to political resources are critical in memory studies. As Hoelscher and Alderman (2004) emphasised two decades ago, people do not recall the past for its own sake, they use it to support different objectives and agendas. Less-privileged groups have been increasingly making use of memory to challenge their own subordination (Hoelscher and Alderman 2004, 349). In this light, a growing body of research has explored how individuals and communities in Latin America build resistance through remembrance. As Lazzara (2017, 14) reminds us, interest in memory studies in this region arose first and foremost out of political activism, where memory ‘has become a battleground and a battle cry’. Hence, in the aftermath of intense political violence, caused by military dictatorships and civil conflicts, memorial landscapes in Latin America are often considered as sites of struggles and conflicts.

Building on De Certeau’s (1980) chief concept, Penglase has for instance stressed how neighbourhood memories could serve as *social tactics.* In Brazilian favelas, histories, memorials and statues are used to resist the neighbourhood’s invisibility and criminalization, without directly challenging those in power: ‘by remembering and forgetting […], by refusing to allow the meanings of the hillside to be determined by more powerful forces’ (Penglase 2014, 175). Similarly, looking at memorials commemorating victims of the so-called ‘war on drugs’ in Mexico, Alonso and Niensass show that in a context of ongoing violence, they are not just symbols but tools for action, advocacy and social transformation (2022, 363). The authors suggest that these alternative commemorative spaces illustrate what James Scott (1985) conceptualizes as the ‘weapons of the weak’: the less visible and less organized forms of ‘everyday resistance’. Alonso and Niensass use the notion of ‘memory activists’ to describe memorial entrepreneurs who are not only concerned with how to remember the past, but also with how to challenge ‘premature calls for closure and ambiguous claims of responsibility’ (2022, 356).

In Colombia, memory and resistance have also generated wide debate these last two decades. In part because of significant institutional changes, the country experienced a memory boom at the beginning of the 2000s (Grisales-Arenas 2012; Lazzara 2017). The 2005 Justice and Peace Law and the 2011 Victims and Land Restitution Law both contributed to empowering victims of the conflict and participated in a surge in memorial work. The first law paved the way for an acknowledgment of the culpability of paramilitaries and their collusion with the state; the second led to the creation of the Centro Nacional de Memoria Histórica (National Centre for Historical Memory, CNMH) which published various historical accounts of the armed conflict. Numerous grassroots initiatives also appeared in the memorial landscape, many of them embodying the idea of resistance. The CNMH’s report on the city of Medellin contains a whole chapter on resistance and memory, describing how music, art, festivals and everyday practices constitute mechanisms of resistance (CNMH 2017). Street art and the broad hip-hop culture are for instance presented as key elements in this context. The control of public space is critical in the criminal governance of Colombian’s cities. In this context, activities organized by the community such as marches, soccer games, meetings, carnivals and festivals are important forms of resistance. They revive collective organization; they encourage the reclaiming of residents’ rights to their city; they challenge invisible borders set by criminal groups.

Finally, Colombia has been the theatre of a high level of political violence, including extrajudicial killings, as well as connivance between public forces and illegal actors, generating deaths, displacements and disappearances. Despite the 2016 peace agreements between the government and the Revolutionary Armed Forces of Colombia (FARC), these issues are still the source of many tensions. The challenges related to the implementation of the peace deal reflect the fervent opposition between those who consider state exactions as mere collateral damage in the securitization of their country, and others who call for a stronger acknowledgement of the state’s responsibility for the violence.

### Right to the walls, right to memory

When Penglase conceptualizes memorial practices as social tactics, he stresses their phantasmagorical dimension, showing how only resident sharing the same history can recognize their meaning. For him, they offer residents a way to resist homogeneous discourses about the place where they live. They also provide means to confront social invisibility and to present their barrio as the product of their own labour. He emphasizes how memorial processes turn the urban landscape into a repository for their stories. Street art offers rich insights when exploring these historical records, as it contributes to unearthing the phantasmagorical. Yet, Vogel et al. (2020) point out that it is often overlooked, even though it represents a valuable source of knowledge about societies in transition, especially in their margins. For them, murals and graffiti are ideal tools for examining perceptions of both peace and conflict, and different versions of histories (2020, 7).

Colombia is again a critical case here, as its main cities have a relatively liberal approach to the use of their walls by street artists. As Grunow (2019) explains, while its capital Bogota had long had an active street art scene, the last decade saw increasingly tolerant policies, turning the city into a mecca for urban artists from all over the world. She adds that the peace negotiations of 2016 sparked an explosion of street art with conflict-related themes, offering politicized, subversive and differentiated representations of the past and present (2019, 42). In this context, street art is a practice mostly born in the margins, and often spread through informal communication circuits (Grunow 2019; Silva 1987). This was initially the case in Medellin, when the street art scene was flourishing. It offered commemorative and political space in a city where community discourses were often erased by hegemonic state narratives about its ‘miraculous’ transformation (Naef 2020; Samper and Marko 2015).

Facing hegemonic discourses on their city, memorial entrepreneurs resist by mobilizing underground memories. Pollak (2006) defines them as belonging to minorities and dominated cultures. He sees underground memories in opposition to official and national memory. Likewise, rather than grand narratives, Grisales-Arenas (2012, 176) associates them with everyday artefacts, murals, family altars, showing how they serve as ways of resisting armed groups and of re-establishing everyday life. While Pollack emphasizes the oppressive and homogenizing dimension of national memory, he nonetheless acknowledges that domination does not always imply a divide between official and underground memory; it does not necessarily refer to the opposition between a dominant state and a subordinated civil society. Accordingly, this article will describe some of the tensions generated by the confrontation between underground memories and official discourses, but also competing memories within marginal communities themselves. Conflicts can for instance arise when underground memories spread into a larger arena. As Pollak (2006) commented, when these memories reach the public sphere, others may seize on them and use them for their own ends.

As the next section will illustrate, some murals and memorial practices were indeed widely featured on the global stage. After being diffused in the tourism sector, the international press, social media and judicial processes, they somehow travelled from the underground to the global. The street art scenes in Medellin and Bogota, for instance, are now one of the most popular tourist attractions in these two cities. As in Belfast and Cairo (Vogel et al. 2020), graffiti tours have emerged in Colombia, helping to establish the creative dimension of its main cities and getting to grips with its violent past. Previous research in Latin America has also revealed how tourism can offer a voice to locals; how it can serve as a vehicle for political action and resistance (Naef 2016; Dürr, Jaffe, and Jones 2020; Freire-Medeiros 2011). In Rio’s favelas for instance, some guides use tourism to criticize gentrification, the lack of social mobility and the consequences of state armed interventions (Dürr, Jaffe, and Jones 2020). In Colombia, and particularly in Medellin where these tours developed in some of the *barrios populares*, tourism was frequently associated with protests and resistance. However, the success of street art tourism brought about the globalization of some of these underground memories; it also significantly reshaped the narratives and representations that they initially carried.

## Walls of resistance

The murals and projects described in this section were developed from 2018 onwards, and they are all associated with recent traumatic events. A brief historical contextualization is thus required to understand the possibilities they offered, but also the tensions they generated. A controversial figure from the Colombian army, associated with some of these disputed memories, can serve as a common theme for this historical account. Mario Montoya Uribe was the *comandante* of the national army until he abruptly retired in 2008, following the ‘false positives’ (*falsos positivos)* scandal.

‘False positives’ refers to the systematic killing of thousands of socio-economically disadvantaged civilians by the army, which presented them as guerrilla fighters lawfully killed in combat (Gordon et al. 2020). Between 2002 and 2008, the army and the ministry of defence offered incentives such as financial rewards and leave days for units achieving high body counts, thus encouraging these extrajudicial killings. As a journalist from the *Guardian* recently commented, what made this case so shocking was ‘the sheer banality of the motive: thousands of civilians were murdered so that the soldiers who did the killing could get more holiday, or a large bonus’ (Palau 2020). The case was brought to light by a group of mothers from Soacha, in Bogota’s outskirts, who managed to trigger media interest in the disappearance of their sons: these young men were found months later in a mass grave far from their homes and were falsely designated as guerrilla soldiers. The story captured the attention of national and international media, although so far, legal action has remained limited.

Before he retired, Montoya Uribe was widely celebrated as a military hero, especially by the *uribistas,* the hard right-wingers of Colombian politics led by the former president Álvaro Uribe Vélez (2002–2010). A few months before the false positives scandal exploded, he masterminded the *Operación Jaque* that brought about the liberation of fifteen hostages held by the FARC, among them the former presidential candidate Ingrid Betancourt. General Montoya Uribe was also famous for being in charge of the *Operación Orión* that took place in October 2002 in the *Comuna Trece* (the commune 13), a peripheral district of Medellin. It was considered as the largest urban military operation conducted in Colombia (CNMH 2015); the objective was to expel the *milicias* (linked to the guerrillas) who were controlling some of the city’s outskirts. This operation was part of Álvaro Uribe Vélez's democratic security policy and was heavily backed by paramilitary forces. It was hailed by the authorities as a triumph of the state over delinquency. Human rights associations, however, stressed that it also left many civilian casualties. This first quote from a former member of the *Bloque Cacique Nutibara* (BCN) paramilitary unit illustrates the violence of *Orión*:

We managed to reach the middle and attack them [the guerrillas]. There were people raising white rags … There was also a helicopter firing. Innocents were asking for peace and orders from our superiors were to keep on going. (Interview with a former member of the BCN paramilitary unit, October 2019)

The second quote indicates the low value placed on the unknown bodies that were buried in an industrial dump after *Orión*:

Only people who died from the confrontations and whom nobody would claim. They were considered as ‘unknown’ or guerrilla. Pickups would climb the hill with the bodies hidden in the dirt. (Idem)

This site is now notorious as the *escombrera* (literally ‘the dump’); it is considered by some as the largest mass grave in Colombia. Moreover, many believe that if the remains of guerrilla soldiers are hidden there*,* other ‘undesirables’ lie there too: petty criminals, mentally ill, drug-addicts, human rights defenders or neighbourhood leaders, many of whom disappeared in the years following *Orión*. The number of bodies buried in the *escombrera* is a subject of debate, although the fact that people disappeared in this area is widely acknowledged. The Special Jurisdiction for Peace (SJP) listed 435 documented cases of possible forced disappearance in the *escombrera* (Ávila Cortés 2021), while some human rights advocacy groups have implied that it may contain as many as 500 bodies. A search process was initiated in 2015, but the possibility of finding remains has been jeopardized by the fact that the dump remained operational until 2010 (and is still in use outside a designated search perimeter). Today, bodies are believed to be buried too deeply under the rubble to be excavated.

While operation *Orión* was seen as a success by some, it nevertheless left traumatizing memories for many others, especially among the population of the *Comuna Trece.* Important commemorations take place every year in October and *Orión* was often represented in the street art scene that developed some years later. Likewise, the false positives scandal triggered reactions in the public domain, where it was depicted on murals and through protests.

### False positives: erasure and globalization

**Figure 1.**
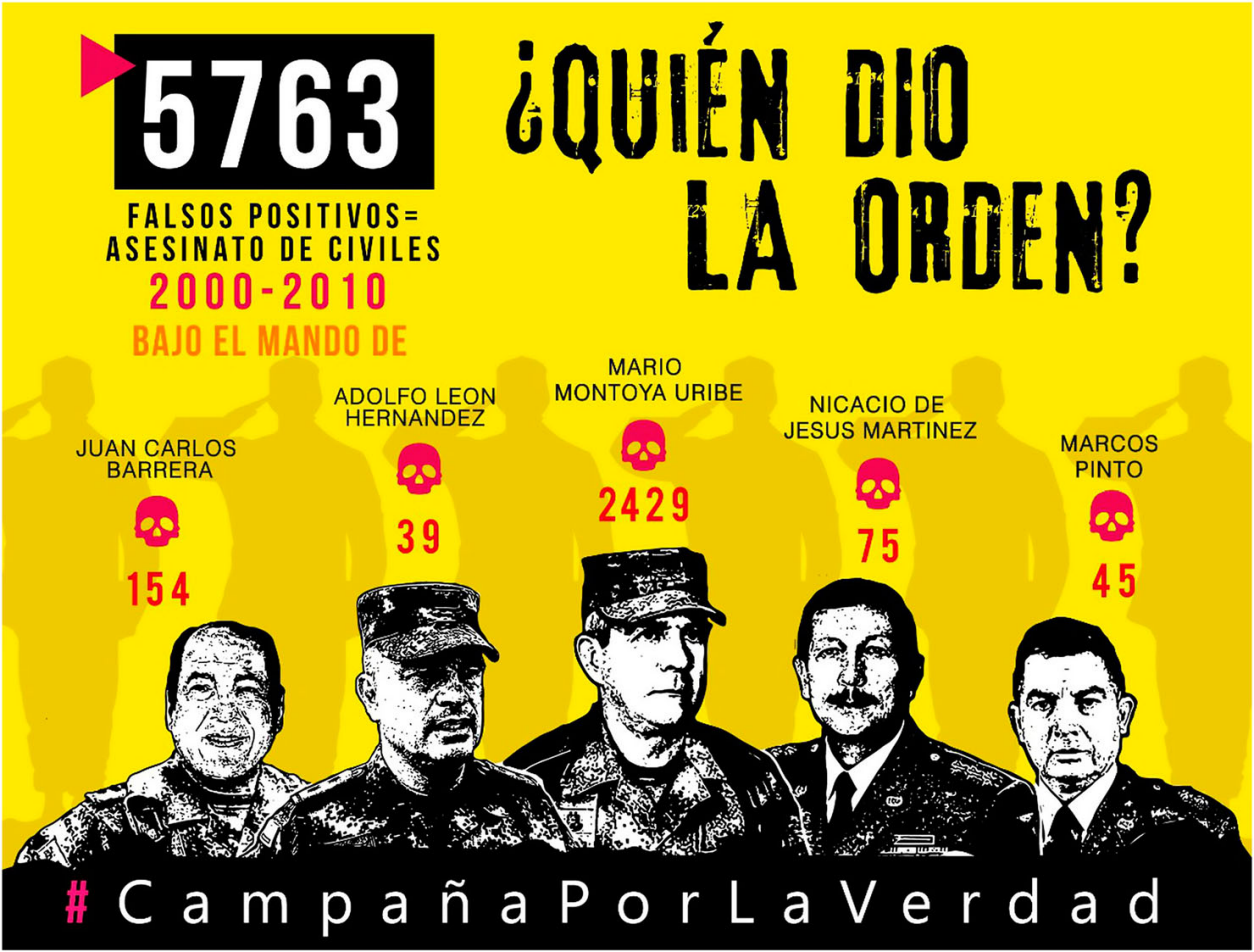
The first version of the mural denouncing the false positive scandal in Colombia (MOVICE, October 2019).

On 18 October 2019, near Bogota’s military school, the army covered up a freshly painted mural (Figure 1). It represented five high-ranking officers and the statement ‘Who gave the order?’ *(¿Quién dio la orden?).* Above each of these military figures was an estimation of the false positives cases that occurred in their zone of authority, accounting for 5763 deaths in total. One of these officers, Montoya Uribe, had the highest number: 2429 cases. The mural was an initiative undertaken by several collectives, among them the National Movement of Victims of State Crimes (MOVICE), which denounced censorship and tweeted a photograph of the military covering up the mural. The hashtag *#QuiénDioLaOrden* promptly went viral. In the days that followed, the concealed mural was replicated on posters, first in Colombia, and then in several European cities. It was also featured in protests, reproduced on tee shirts and facemasks, and used by people as their social media profile. As time went by, other army officers suspected of involvement in the false positives case were progressively added to the picture and the number of cases was updated. In 2021, the number of dead was 6402, according to an estimation of the SJP (Delcas 2021).

The mural’s message gained even more resonance after one of the officers pictured, General Marco Pintos, requested that MOVICE withdraw the image and acknowledge its ‘mistake, negligence, error, or misrepresentation’ (El Espectador 2021). After several legal developments, the constitutional court nonetheless declared the mural to be an expression of free speech and defended its right to be replicated. As the regional daily *El País* said in its headline in November 2021: ‘political street art won one of its most important victories in Colombia’ (Osorio 2021). The mediatization of the mural reinforced a transnational movement denouncing the false positives scandal. At the end of August 2021, around the International Day of the Victims of Enforced Disappearances, its colours and the hashtag were used in an international campaign called *Los 6402.* In many cities in Europe and the Americas, performances were organized with rubber boots as a central symbol, representing the disguising of false positives as guerrilla soldiers. Finally, in April 2022, a first ‘public hearing of acknowledgment’ took place in the Colombian city of Ocaña. Ten members of the army (among them a general) publicly recognized their role in these extrajudicial executions, in the presence of victims.

The false positives mural illustrates the evolution of contested representations of state violence. The story of the false positives, brought to light by the mothers of disappeared persons in the outskirts of the capital, progressively gained national notoriety. When the army failed to prevent an image denouncing its impunity from travelling further, it contributed to the globalization of the message. People used social networks as powerful tools for publicizing these contested images and narratives. The obstacles raised by army officials to neutralize these memorial representations increased their visibility. The contested memory of false positives thus travelled from the underground to the global, and by 2021 was reaching audiences in some of the world’s main capitals. Moreover, as the progressive acknowledgment of the state demonstrates, the erased mural contributed to visualizing a process of resistance instigated fifteen years ago by mothers of the disappeared.

### The live gallery: memories of the barrio

While the example I present above addresses memory on a national scale, since false positives were found in mass graves in various areas of the country, the memorial representations described in this next section belong to the history of specific neighbourhoods. They do not implicate high-profile figures of the army, nor were they featured in the national press. They are micro-stories of residents’ victimization in the urban margins of Medellin. They are however shaped by a larger political context, especially the paramilitarization of some parts of the city following the operation *Orión*. These stories have been assembled by a collective named ‘Agroarte’; they are represented on the walls of what the collective terms as an outdoor museum and the ‘largest painted cemetery in Latin America’. Agroarte was born in 2002 as a process of resistance to the *escombrera.* Its members have been conducting memory work in Medellin and Colombia, bringing together artistic practices (such as rap and street art), urban agriculture and youth integration. The project described here, launched in 2018, is called the ‘Live Gallery’ (*Galeria Viva*) and is situated in the cemetery of *La América* in the *Comuna Trece.* After being practically abandoned for several years, this site was placed under the administration of *Campos de Paz,* an organization tied to the Archdiocese of Medellin. As Agroarte members explained, to be able to paint on the walls of this cemetery, they first needed the acceptance of the local community. Later, hey negotiated with employees working in the cemetery but never with representatives of *Campos de Paz*:

We started to paint the walls and organize activities for the community in the cemetery. We got a kind of general permit from the local administration, but we never talked to *el padre* (the father) … We talked to the community and some administrative employees who usually support us. And now that the space is occupied … they don’t say anything. (Interview with community leader, February 2022)

Some of the early murals illustrate this arrangement, like the portrayal of *El Tigre* (the tiger), which represented the local gravedigger, an ally of the collective who died in an accident shortly after the project was launched. Another depicted the Virgin of Guadalupe. Although Agroarte does not have any Christian links, it nevertheless wished to show respect for the believers in the community. Generally, the murals are not permanent, but their themes are very often centred on the violence in the barrios

**Figure 2.**
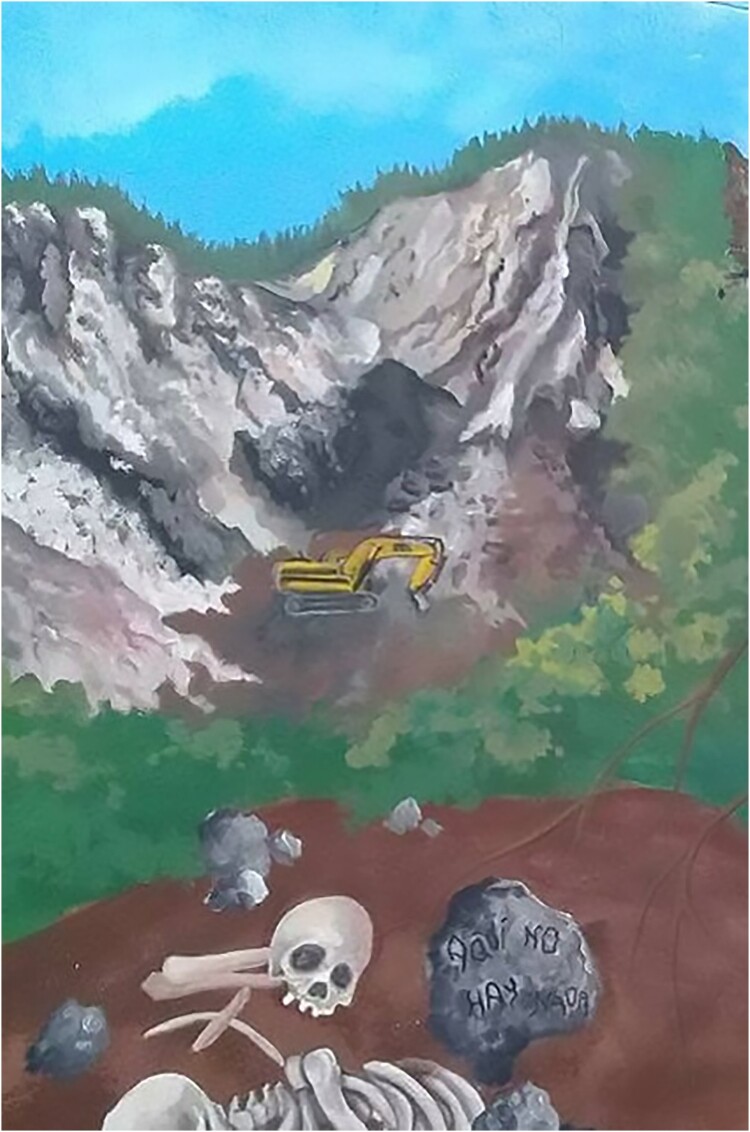
A picture of the *escombrera*. The truck in the background illustrates the fact that the dump is still partly operational despite the bodies buried there. The inscription on the stone says: ‘There’s nothing here’ (*Aqui no hay nada)* (Author, January 2019).

**Figure 3.**
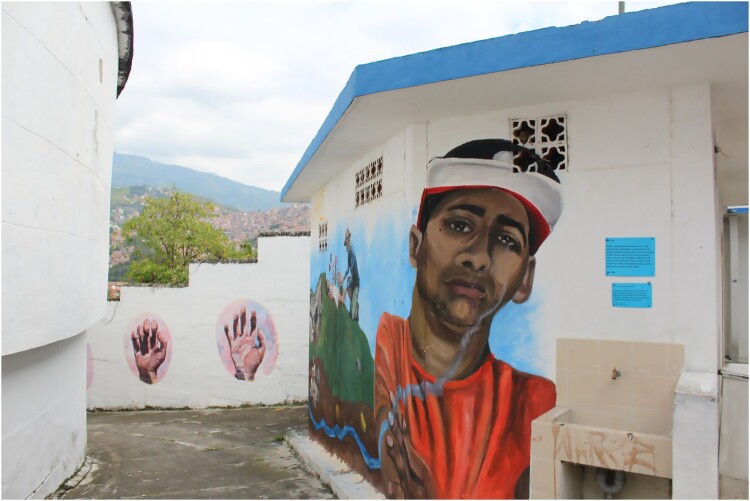
One of the many portraits of teenagers killed in Medellin (Author, January 2019).

Even today, the *Comuna Trece* remains one of the most violent districts of the city. With its strategic position on the Colombian narco-road, *La Trece* has one of the highest rates of homicides, forced displacement and extortion. After *Orión*, this disputed territory came under paramilitary control until demobilization took place between 2003 and 2006. The failure of the demobilization process eventually led to the integration of many of these former combatants into street-gangs colloquially labelled *combos*. Hence, several murals in the cemetery depict local histories, for instance by portraying young men assassinated by gangs during the last decade (Figure 2). However, some murals have a broader scope, illustrating the political violence in the country. They often feature harsh criticism of the politics of the former president Uribe Vélez, including the infamous *escombrera* and the forced disappearances that occurred during the post-*Orión* period (Figure 3).

Several *rostros* (faces) are painted on the walls surrounding the cemetery: young hip-hop artists killed recently, but also people who are alive and significantly engaged in memory work and human rights defence. One of the murals represents a teenager shot dead in 2017 after he used a path he was not supposed to. His mother explained that after her son was killed, the family had to leave the *Comuna Trece* in a hurry*.* The perpetrator lived in the same neighbourhood and the prosecutor told her that if they lodged a complaint against him, they would have to leave to avoid retaliation. Referring to the *escombrera,* she added:

There was an invisible frontier that cost the life of my son two years ago. The war took another one twenty years earlier. […] They killed two of my sons, but I know where they are. There are currently many mothers who do not know where their children are. (Interview with resident, October 2019).

Several months after the drama, Agroarte contacted her about their project. She sent them a photograph of her son, and later, on a rainy afternoon, they took her to the cemetery: ‘When we arrived, [we saw that] they had painted a mural for him! We performed a ritual of planting for him … giving life through a plant’ (idem).

Urban gardening is indeed central to Agroarte’s approach to memory work. The collective has been transforming numerous waste dumps all over the city into garden plots, symbolizing their resistance to the *escombrera*. As the leader of the collective stated:

With a group of eight *doñas*, mothers or grandmothers of *desaparecidos*, we started to plant medicinal plants in areas close to the *escombrera*. We wanted to create places in the city where people met and did productive work … work related to the protection of the territory and a work of memory. We were the first to talk about the *escombrera*. We brought it into public view. (Interview with community leader, October 2019)

From 2014 to 2018, on 16 October, as a reminder of *Orión*, Agroarte also organized a memorial and artistic performance called ‘Grammatical Bodies’ *(Cuerpos Gramaticales)*. Dozens of people, many of them relatives of *desaparecidos*, buried themselves up to their chests for six hours, in strategic places in Medellin. The objective was to physically and symbolically root themselves in the ground in order to claim their right to their territory. It was also a silent protest against the forced disappearances and the criminal control of their neighbourhoods. After several events in Medellin and other Colombian cities, the performance began surfacing in other parts of the world. In Guernica and Barcelona, it highlighted forced disappearances, but also problematics of migration and displacement.

The collective promotes local histories through their live gallery of murals and addresses memorial issues on a larger scale with the Grammatical Bodies commemoration. The target audience in the Live Gallery project is the neighbourhood, even if international visitors are often welcomed. With the murals*,* the collective enhances the significance of the cemetery of *La America* for the community; it contributes to unearthing this previously abandoned place. Nowadays, performances and concerts are organized, and gardening sessions bring together different generations. Teenagers learning rap and street art also work in the gardening plots with the elders pleading the cause of their disappeared relatives. Agroarte thus revives underground memories to foster a sense of place in the *comuna.* Live Gallery and Grammatical Bodies are different in their scope, but their objectives converge: memory work is a means of resisting oblivion, but also of reclaiming public space.

### Graffitours: the depoliticization of the walls

One kilometre away from the cemetery of *La America,* another sector of the *Comuna Trece* has seen the development of an important movement of urban art. Every year of the last decade, new colourful murals and breakdance performances have emerged at the site known as *Las Escaleras^.^.* These outdoor electric escalators were initially built to improve the mobility of residents. Due to their innovative nature, but also to the surrounding street art, they turned into a very popular tourist attraction. This urban project is now a posterchild of Medellin’s social improvement, but it has also become very controversial within the community (Naef 2020, 2023). Gradually, after the creation in 2011 of the *Graffitours* by two local hop-hop artists, who presented the street art of the barrio, what began as a relatively underground touristic and memorial practice turned into mass tourism. According to a departmental census, *Las Escaleras* welcomed 436,395 visitors in 2019, of whom 70% were international tourists (SITUR 2019). In 2020, the ‘Tourism Network of the *Comuna Trece*’ identified approximately 450 guides in the area.

**Figure 4.**
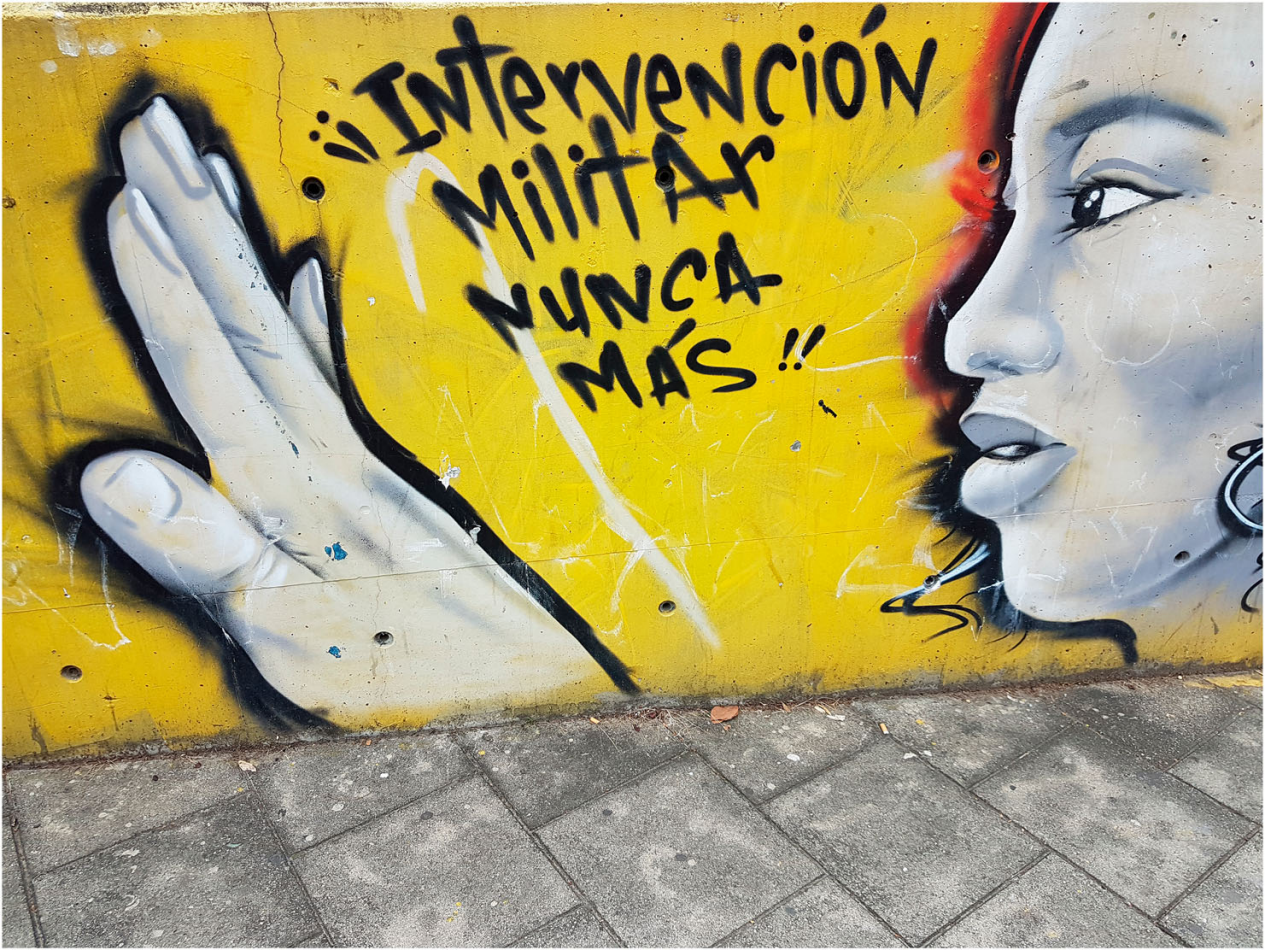
Mural in the *Comuna Trece* in: ‘Military Interventions, Never again.’ (Author, October 2017).

As with other tours in Latin America (Dürr and Jaffe 2012), close contacts and conversations between tourists and residents are central. Initially, the discourse of tour guides was often politicized, using street art to illustrate the violence of the *Comuna Trece*. Guides directed their criticism towards the intertwinement of the state and illegal groups, the criminal governance of the barrios and the traumatic consequences of armed operations. Some guides were also very critical of the escalators themselves, implying they were useful primarily for the branding of the city, rather than for the residents. However, with the increase in tourism entrepreneurs, and especially with the arrival of many guides from outside the barrio, the discourse shifted. Instead of criticism, some guides centred their narrative on *La Trece* as a showcase for Medellin’s miraculous transformation, praising the way *Las Escaleras* had positively changed residents’ lives. Others emphasized the creativity and resilience of the *comuna.* This dynamic can be seen in the changing topics of the street art itself, with murals representing violence, such as images associated with (para)military operations (Figure 4), giving way to thematic associated with landscapes or animals.

Naturalistic or animal representations can of course be as political as more realistic portrayals of violence. In a mural produced several years ago next to the escalators, a collective depicted *Mariscal,* another traumatic armed operation that occurred in May 2002, as an elephant and a black hawk. The first symbolized the importance of memory, the second, the helicopters used during these operations. Moreover, murals in *La Trece* are sometimes associated with indigenous stories, in which animals often have a strong political meaning. Yet, as Vogel et al. (2020) emphasize, commissioned pieces of street art abound on Medellin’s walls, together with Instagram and Twitter handles for viewers to follow the artists. They add that these murals often assume non-political functions: ‘some are to beautify touristic neighbourhoods; some are commissioned for commercial reasons to advertise hostels and restaurants’ (2020, 13). According to *El Tiempo,* artists themselves occasionally pay to paint a mural in the *Comuna Trece*, for instance when they need publicity for launching a new project (El Tiempo 2021). In such cases*,* neighbourhood committees define rules and approve proposals, demanding vibrant colours, peaceful messages and a positive picture of the community (Vogel et al. 2020, 13).

The success of tourism at *Las Escaleras* led to the involvement of local gangs, through the extortion of tour guides, street artists and business owners (Author 2022). Promoting critical discourses on illegality and violence, while at the same time financing gangs with tourism profits, became a concern: ‘How are we going to propose a tour on social transformation if a percent of the money that you are paying is going to these illegal groups?’ (Interview with tour guide, August 2020). Another tour guide implied that gangs*,* like neighbourhood committees, controlled the walls of the barrio: ‘even to paint a wall you have to ask for permission and you have to pay the *delincuente*, because you can't paint a wall anymore without asking for permission’ (Interview with tour guide, March 2021). For some residents and tourism entrepreneurs, the take-over of tourism by illegal actors also increased local drug trafficking and prostitution. A community leader involved in youth violence prevention for instance severely criticized this phenomenon:

One street away from where *Graffitours* end, *niñas* (young girls) sell themselves to foreigners. Something you would not have seen before in the *comuna.* One street away from the *Graffitours* you can find all kinds of drugs. So you have all these cool dynamics helping to understand the history, and afterwards you go to smoke *un bareto* and you lose everything you came for. (Interview with community leader, March 2021)

Echoing this statement, several interviewees stressed that the important economic impact of tourism contributed to weakened memorial spaces and practices. The interviewee quoted above added that in her opinion some people in the tourism business still had a political objective: ‘To talk is a political action. To claim: ‘Here they killed us; here they buried that many people.’’ However, for her, the explosion of activities related to tourism – for instance the numerous breakdance groups performing around *Las Escaleras –* had hardly any connection to politics. Many were only in it for the money. She added that the same could be said of some tour guides: ‘It prevents the construction of memory. One goes to listen to a Venezuelan or an armed group member guiding a tour, narrating stories to people and one knows that he is a *delincuente*. You know that he will make you pay if you tell your own story.’ Some residents, especially in the surroundings of *Las Escaleras*, felt they were being dispossessed of their history by the inaccuracies and inventions propagated by some of the new (and often outsider) tour guides. For many artists and residents, the practice had become a product:

The graffiti are very attractive, but they do not speak to me. […] People know that in this business there are a fair number of foreigners, so random people [tour guides] stand there and pay the *vacuna*^1^*,* telling the wrong things, false histories … And they [the foreigners] take pictures and leave. This is not a site of memory. (Interview with a resident, March 2020)

I feel it as a cool process and that it has good things, like how the people started to manage entrepreneurship … how the *señora de la cremas* (the ice-cream lady) manages to support her family. But I think we lost what was at its heart; it turned into a business to show graffiti and to charge [the visitor]. (Interview with a resident, March 2020)

Parents won’t tell kids to study but to go to the *viaducto* because they know that a 5- or 8-year old kid will manage to earn 50’000 pesos in less than a half-day. The girls are turning to prostitution. This is exploding! […] Presenting our arts, something that started de *manera bacana* (in a cool way) … at the end evolved into something else. (Interview with an artist, June 2020)

A striking example among the many unverified facts and urban legends incorporated into the touristic discourse is the frequent integration of the drug lord Pablo Escobar, who had little or nothing to do with the history of *La Trece.* The example of Escobar allows me to conclude this exploration of Medellin’s walls, and the memories and histories they carry. Nowadays, Pablo Escobar is associated with many contested representations in popular culture, fashion, tourism and unsurprisingly in some of Medellin’s murals. Many of them are situated in a neighbourhood initially named ‘*Medellín sin tugurios’* (Medellin without slums), but colloquially labelled as the ‘*Barrio Pablo Escobar*’. In the eighties, the former boss of the Medellin cartel, in keeping with his political aspirations, offered housing plots and construction material to hundreds of families living on a municipal landfill.

Recently, this neighbourhood also began attracting tourists, although in far more limited numbers than in the *Comuna Trece*. Nowadays, visitors are interested in the history of this peculiar urban development, but also in getting selfies in front of murals that have already been largely featured on the web. Besides the many representations of *el capo*, murals also portray other members of the cartel. One is dedicated to Popeye, his main hitman, and even Terremoto, the horse of Escobar’s brother, is featured. Roberto Escobar, the brother of Pablo and former bookkeeper of the cartel, opened a controversial museum on both himself and his brother in another area of Medellin (Naef 2018). He also contributed to an informal exhibition in a small barbershop in the *Barrio Pablo Escobar*, providing some photographic archives with intimate insights into the Escobar family. The barbershop also sells Pablo souvenirs: mugs, caps, key rings, stickers, etc.

The murals in *Barrio Pablo Escobar* are a significant contrast to the ones exposed in the Live Gallery, which focus on victims of the violence and on human rights defenders. They also differ from the images displayed in the *Comuna Trece,* where portrayals of the cartel members are unlikely to be produced. However, they follow a similar commodification dynamic: souvenirs are available, and in both neighbourhoods, Pablo Escobar is widely present in the merchandising. Moreover, nowadays tour guides and companies need to contribute financially when they bring in tourists. When I mentioned extortion in the *Barrio Pablo Escobar*, one of the neighbourhood leaders nonetheless strongly asserted it was not the same as in *La Trece*:

No, no … It is not a *vacuna*, because here one does not charge any *vacuna*. It is just that when people want to know about the barrio, they need to leave a little contribution for the maintenance of the murals. (Interview with community leader, February 2020)

She added that when tourists arrived, she or someone else from the barrio collected money to buy paint or other items. The exact nature of this contribution is murky, and if tourism were to increase in the years to come, the example of the *Comuna Trece* could doubtless bring some insights into its management in such a barrio, and more broadly in informal areas associated with past, and sometimes ongoing, violence.

## Politics, aesthetics and the (un)earthing of violence

The murals described in this contribution are diverse. They are produced by actors guided by various objectives spanning aesthetics and politics, and cover fields ranging from the defence of human rights to the development of international tourism. Consequently, their everyday reverberation affects their socio-political contexts in different ways. The memories they unearth are often contested, and their journeys imply various possibilities and limits.

Examining the modalities of (un)earthing political violence provides a fertile ground for scholars in memory studies for deconstructing dichotomies such as official/underground, state/community, perpetrators/victims. The short-lived murals in Bogota certainly promoted the progressive recognition of false positives in Colombia and elsewhere. Victims could challenge the closure that official narratives may express. However, in keeping with Pollack’s conception of underground memories, I suggest that if national memory can be homogenizing and oppressive, competing representations also arouse within communities. Murals and other commemorative projects presented in the tourism sector can represent a stand for peace and provide a deeper understanding of local contexts. At the same time, they can also homogenize and simplify the history of places plagued by violence.

Some residents felt for instance that the touristification of street-art in *La Trece* depoliticized its messaging, by expunging the complexity of its history. Many interlocutors agreed that murals allowed the diffusion of *micro-relatos* (micro-narratives) in a context dominated by what they sometimes referred to as totalitarian memory. However, with the globalization of these underground memories, a significant number of them criticized the narrow focus of current memorial discourses: ‘The memory of the territory is not only about the conflict. What about the *convites*^2^ and other organizational processes?’ ‘It seems as if the *comuna* was invented along with the hip-hop bands. There is certainly a will to recuperate certain memories, but nobody talks about those who arrived in the 50’s. About their struggles and their resistances.’ (Interviews with residents, March 2021) Moreover, the recuperation of tourism by street-gangs in the *Comuna Trece* demonstrates how some memorial discourses and representations are entangled in the violence of the barrios. With the development of tourism, (un)earthings in the *Comuna Trece* are displayed in the expression of violent memories, but also in their erasure. The *Barrio Pablo Escobar* illustrates the contested reverberation of violence in Colombia. While portrayals of Pablo Escobar and his cohort dominates the local street-art, the drug-lord’s heritage is hotly contested in the second city of the country. Nevertheless, his image lives on in the barrio. Agroarte, the collective involved in the Live Gallery, started to develop a similar project in this neighbourhood. After discussions with residents*,* they painted two murals next to the main portrayal of Pablo Escobar:

We wanted to create another outdoor museum to recount an alternative history of this barrio: the history of the way people self-built this neighborhood. Pablo is of course part of this history but is not as central. We wanted to demystify this. (Interview with community leader, September 2022)

**Figure 5.**
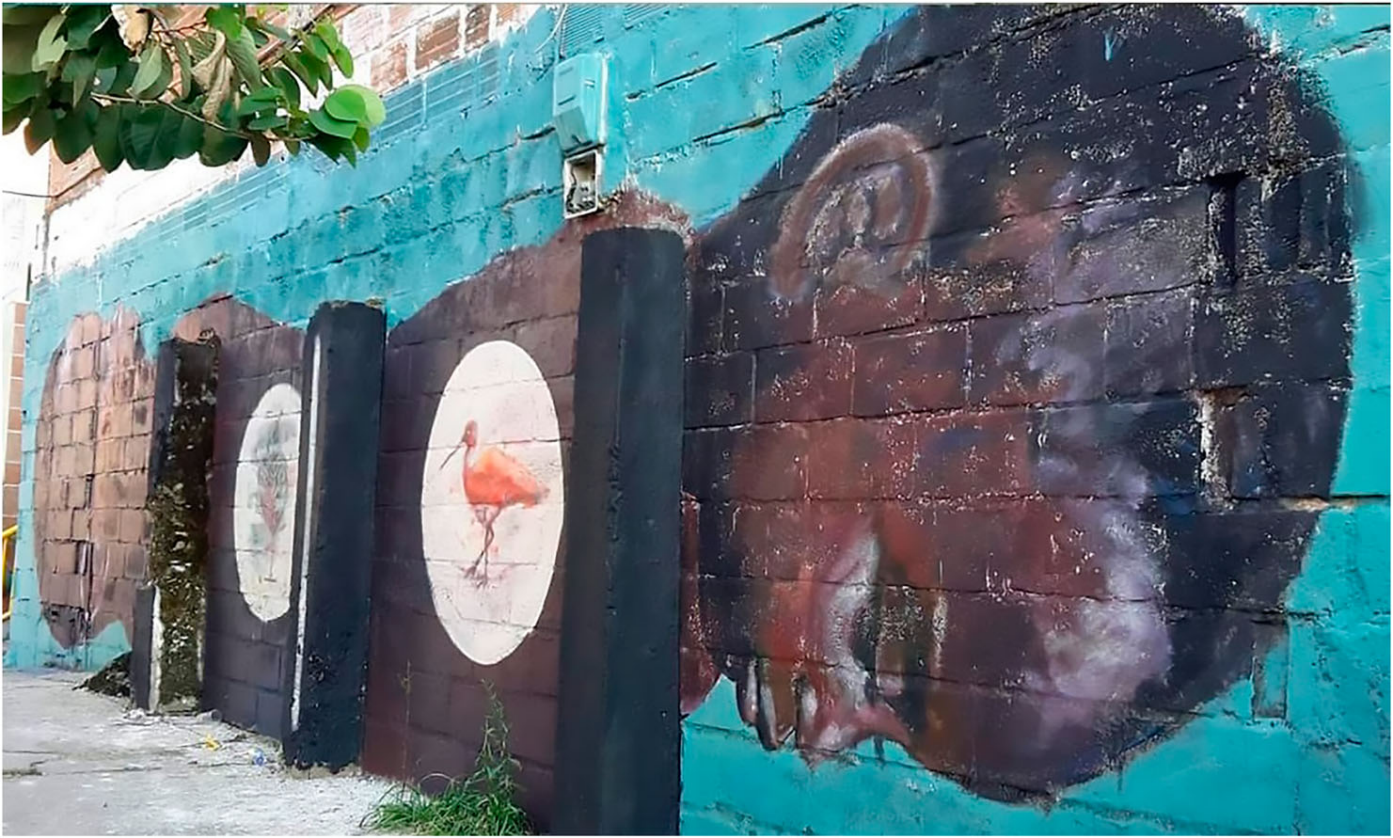
A mural of an afro descendant kid painted by Sr. OK (Agroarte, 2017).

**Figure 6.**
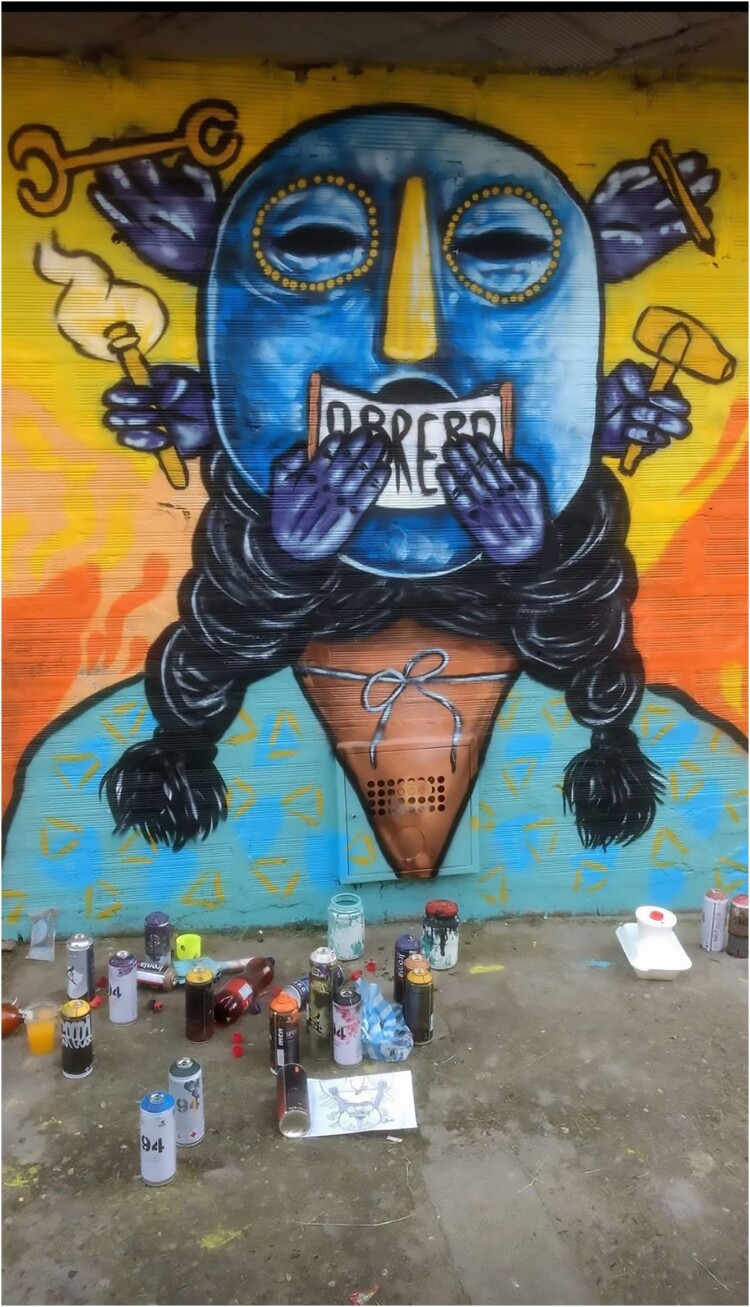
A mural painted by Joma representing a worker *(obrero)* (Agroarte 2018).

**Figure 7.**
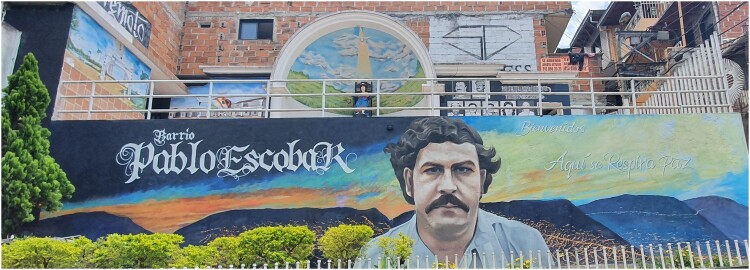
The area in the barrio shown to tourists with a mural of Pablo Escobar and the inscription: ‘Welcome, here one respires peace’ (Author 2022).

The collective worked in collaboration with two *grafiteros* of the city. Firstly, Sr. OK painted the face of an afro descendant kid just next to the main mural of Pablo Escobar (Figure 5). Secondly, on the other side of the street, Joma realized another mural representing a worker (Figure 6). Agroarte then organized a concert next to these two new murals to attract people from adjacent neighbourhoods. Finally, the collective arranged visits at their Live Gallery to enable people from the barrio to discover their project: ‘I think that when we brought residents of *Barrio Pablo Escobar* to the Live Gallery, it showed them what they could do in their own barrio’ (idem). However, the project also inspired the local gang who eventually covered up the new murals to develop its own conception of street-art. The *combo* controlling the area commissioned a street-artist from Bogota, who painted a representation of the *Casa Monaco*^3^ instead of the young afro descendant kid’s picture. They also repainted the main mural of Pablo Escobar (Figure 7) and replaced Joma’s mural with another representation of the drug lord.

The *combo* used the information they got from our own project and centered the narratives on Pablo again. We decided to stop painting murals in these areas after that. We were not willing to collaborate with the *pillos* (criminals) in any way. (idem)

Hence, reproducing the Live Gallery in *Barrio Pablo Escobar* never took place. The local gang considered that representations of the boss of Medellin’s cartel were more appropriate, especially with the recent appearance of tourists eager to discover this unusual barrio.

Finally, the Live Gallery established in the *Comuna Trece* was possible only because of its main protagonist’s legitimacy. The group had known residents for years and could thus unearth some of their most traumatic memories. While the project is now widely accepted in the barrio and most visitors are locals, some murals arouse hostility and occasionally trigger violence. One of the murdered teenager’s murals was for instance attacked one week after it was painted: the neck and the face of the young man had been scratched with a knife.

Beyond their political meaning, the murals examined above also reveal contrasting styles. While some are produced by experienced artists, others feature more naïve and amateur modes of creation. In the Live Gallery, some murals were created by renowned *grafiteros* of Medellin and others by residents with much less experience. Some residents for instance painted themselves their disappeared relative, while others assigned the task to an artist working from a photograph. In this case, the micro-histories associated with the mural often seem more important than its aesthetic or technical skill. New murals involve a public launching in the presence of relatives and creators. The false positive mural and most of the ones featured on the walls of the *Barrio Pablo Escobar* are also minimalist in style and technique. In the first case, the aim was to create a powerful contrast of colours (black and yellow) to enhance its visibility and impact. In the other, murals are painted by local youth, most of them involved in the gang, who transform some of the walls into a large comic strip on the cartel’s history. Yet, as the dispute described above illustrates, they occasionally commission external artists, like the one from Bogota who painted the most recent murals of Pablo Escobar and his *Casa Monaco.* It is in the surroundings of *Las Escaleras* in the *Comuna Trece* that aesthetics, technicity and skills are the strongest. Nowadays, murals are all produced by confirmed *grafiteros* who use vivid colours to present a lively neighbourhood that reflects the positive messaging of tour guides. Yet, beyond these differences in style, all of the murals have two dominant features. They are first of all ephemeral and usually last no more than a couple of years. Secondly, they all need to be ‘instagrammable’, and have the potential to appear on social networks. Indeed, these are now the main vessels by which these underground memories travel.

## Conclusion

Although it is less overtly visible, memorial processes in Colombia take place in spaces where violence lingers on. In this context, memory work, official or underground, does not automatically lead to greater respect for human rights or democratic values. (Un)earthings provide resources for resistance, but may also create conflicts and dispossessions. The reverberation of political violence in Colombia contributes to peace but also helps strengthen memory conflicts. As this article illustrates, murals depicting violence and the public spaces where they appear can become memorial battlegrounds. It is thus critical to question the dual nature of these battles, as well as the traditional categories of memorial entrepreneurs. These may be scientists, curators or activists, but also *grafiteros,* tourist guides or criminals. As this special issue emphasizes, memorial processes are dynamic and contested. They are fragmented and elusive, transgressive and homogenizing, and sometimes even incoherent, based on intellect as much as affect.

Describing cities as arenas where narrative battles take place, de Certeau insisted on the importance of *lieux de parole* (spaces to talk) for a city to breathe (1980). The murals presented in this article differ in their scope and objectives, but all represent important *lieux de parole.* They allow the diffusion of narratives, symbolically through their aesthetic and their messaging, and materially through the ceremonies, marches, social gatherings or guided tours they generate. The Live Gallery focuses primarily on the neighbourhood: reaching a more global arena and issuing larger political claims are not the project’s principal goal. Yet, while most of the subjects of the murals are stories of the barrio, some of them also concern larger issues. Critical portrayals of the former president Uribe Vélez have a national resonance. They concentrate however on the impacts his policies had in the *Comuna Trece,* as illustrated by representations of *Orión* and the *escombrera.* The mural denouncing false positives is a different case: it originates in a larger collective made up of several advocacy groups, some of them operating at the country level. The national impact of false positives explains how it promptly resonated on a global scale, but the military counterattacks certainly contributed to internationalize these representations. At different levels however, both initiatives share a similar objective: their memorial work is committed to resistance, denunciation and catharsis.

In contrast, while the touristification of street art in both *Comuna Trece* and *Barrio Pablo Escobar* began within the community, in *La Trece* the shift from so-called ‘community tourism’ to mass tourism significantly affected the messaging. Tourism there initially carried a political narrative, highlighting the roots of the violence in the area. In the long run, it nevertheless participated in the empowerment of the structures it was criticizing, prompting several interlocutors to question a practice they considered as having abandoned its political origins. Paradoxically, the success of tourism brought political resources into the barrio, but at the same time depoliticized the narratives and enhanced the victimization of its residents*.* Touristic development in *Barrio Pablo Escobar* is still very limited. The many images of Pablo Escobar and his lieutenants on the walls of the neighbourhood, however, raise the question of their role, between commodification and politics. Further research could examine more thoroughly the perceptions of these murals by both residents and visitors, including aspects such as gratefulness to their benefactors, historical testimonies, pop culture aesthetics and glamorization of violence.

These examples also illustrate some of the challenges of commemorating, denouncing and representing past violence through images. They partly reflect Lazzara’s vision of the memory boom in Colombia, in which he states that despite prolific grassroots, academic and artistic productions, memory work sometimes lacks narrative complexity. For him, it often fails to detail specific causes and forms of violence (2017, 18). Tourism as a memorial practice certainly carries the risk of depoliticizing the debate on violence, since short-term interactions with visitors rarely allow such complexity to be addressed. Moreover, the development of tourism in Colombia is taking place in a context where it is considered as a driving force for peace and reconciliation. Accordingly, tourism actors, public and private, have recently seemed more inclined to promote consensual narratives than to engage in the memorial battlefield.

Finally, despite numerous memorial productions by the Colombian state, such as reports, museums and memorials, several observers have commented on the weak institutional support for neighbourhood initiatives. To challenge the lack of institutional support, memorial entrepreneurs use underground memories as social tactics to carry out everyday resistance. The Live Gallery*,* for instance, provides a space for victims to recall their histories and to provide testimonies without directly confronting the elite. As members from Agroarte highlighted, they negotiated the production of murals in the cemetery directly with the local community and the employees working on site, and not with the clerical authorities. While the mural of false positives directly challenged the state, the political criticism of street art in *La Trece* relied on more consensual representations. Yet, as James Scott underlined, everyday resistance lies somewhere in a continuum between defiance and compliance (1985, 136). Hence, to different degrees, all the memorial projects presented in this analysis challenge state narratives. Through representations of murdered teenagers, suspect military officers and even drug cartel bosses, they raise questions of social justice, impunity, illegality and the dramatic banalization of violence in the country. They broaden the narrative on the recent past, through histories and images that the Colombian state is still reluctant to unearth.

## Notes

1 ‘The vaccine’, referring to the extortion tax throughout Colombia.

2 *Convites* describe collective actions and the solidarity networks that residents use to build informally their neighbourhood.

3 The *Casa Monaco* was one of the residences of Pablo Escobar. It was also one of the main highlights of ‘Pablo narco-tours’, until it was destroyed by the public authorities in 2019.
